# Effects of rogue ryanodine receptors on Ca^2+^ sparks in cardiac myocytes

**DOI:** 10.1098/rsos.171462

**Published:** 2018-02-21

**Authors:** Xudong Chen, Yundi Feng, Yunlong Huo, Wenchang Tan

**Affiliations:** 1 State Key Laboratory of Turbulence and Complex Systems and Department of Mechanics and Engineering Science, College of Engineering, Peking University, Beijing, People's Republic of China; 2 PKU-HKUST Shenzhen-Hong Kong Institution, Shenzhen, People's Republic of China; 3 Shenzhen Graduate School, Peking University, Shenzhen, People's Republic of China

**Keywords:** Ca^2+^ spark, Ca^2+^ quark, anomalous subdiffusion, rogue ryanodine receptors

## Abstract

Ca^2+^ sparks and Ca^2+^ quarks, arising from clustered and rogue ryanodine receptors (RyRs), are significant Ca^2+^ release events from the junctional sarcoplasmic reticulum (JSR). Based on the anomalous subdiffusion of Ca^2+^ in the cytoplasm, a mathematical model was developed to investigate the effects of rogue RyRs on Ca^2+^ sparks in cardiac myocytes. Ca^2+^ quarks and sparks from the stochastic opening of rogue and clustered RyRs are numerically reproduced and agree with experimental measurements. It is found that the stochastic opening Ca^2+^ release units (CRUs) of clustered RyRs are regulated by free Ca^2+^ concentration in the JSR lumen (i.e. [Ca^2+^]_lumen_). The frequency of spontaneous Ca^2+^ sparks is remarkably increased by the rogue RyRs opening at high [Ca^2+^]_lumen_, but not at low [Ca^2+^]_lumen_. Hence, the opening of rogue RyRs contributes to the formation of Ca^2+^ sparks at high [Ca^2+^]_lumen_. The interplay of Ca^2+^ sparks and Ca^2+^ quarks has been discussed in detail. This work is of significance to provide insight into understanding Ca^2+^ release mechanisms in cardiac myocytes.

## 1. Introduction

Ca^2+^ sparks regulate the excitation–contraction coupling in heart muscle [1–3], and are activated by the opening of clustered ryanodine receptors (RyRs) on the junctional sarcoplasmic reticulum (JSR) membrane [4–6]. Recently, the discovery of quarky Ca^2+^ releases (QCRs or Ca^2+^ quarks) due to the opening of rogue RyRs has been shown as a significant Ca^2+^ release mechanism relevant to ‘invisible Ca^2+^ leak’ [7–9]. Here, rogue RyRs refer to RyR channels located near clustered RyRs within a JSR, defined as ‘junctional rogue RyRs’. Although Ca^2+^ sparks and quarks occur spontaneously and concurrently in the cytoplasm under physiological conditions, Ca^2+^ quarks feature different properties from Ca^2+^ sparks, e.g. a smaller amplitude with a high firing frequency and a longer duration [10].

Zima *et al*. [11] demonstrated experimentally that single RyR opening could mediate Ca^2+^ leak but fails to trigger Ca^2+^ sparks when free Ca^2+^ concentration in the JSR lumen ([Ca^2+^]_lumen_) is below a threshold level. On the other hand, mathematical models have been developed to investigate the dynamics of Ca^2+^ sparks and quarks. Sobie *et al*. [12] proposed a model to solve the paradox of [Ca^2+^]_lumen_ to support the existence of rogue RyRs. Models of Ca^2+^ leak from JSRs have been used to characterize differences between Ca^2+^ sparks and quarks [13]. Walker *et al*. [14] included the computational nonspark-based Ca^2+^ leak from JSRs to explain the exponential rise of Ca^2+^ spark frequency. They further presented that Ca^2+^ sparks could be triggered by spontaneous opening of a single RyR in a cluster [15]. A stochastic contact network model of the Ca^2+^ initiation process was applied to realistic RyR cluster structures, which revealed that the Ca^2+^ sparks probability depends on the position of the initial RyR in the cluster. Their works provided insight into the Ca^2+^ release process in the heart and a framework for evaluating functional heterogeneity in populations of receptor clusters under normal and pathological conditions. Lu *et al*. [16,17] investigated ‘non-junctional rogue RyRs’ located away from the release sites and showed their effects on Ca^2+^ waves with heart failure. It suggested that Ca^2+^ dynamics was unstable and Ca^2+^ waves were likely to be triggered when ‘non-junctional rogue RyRs’ were taken into consideration. The variation of membrane potential depolarization was indicated to be dependent on the distribution density of rogue RyR channels, which is important for understanding the arrhythmogenic mechanism for heart failure from the subcellular to cellular level. Sato and Bers [18] used a mathematical model of JSR Ca^2+^ releases to show that single RyR opening at low [Ca^2+^]_lumen_ could not recruit Ca^2+^ sparks from Ca^2+^ release units (CRUs). However, the effects of rogue RyRs on Ca^2+^ sparks at high [Ca^2+^]_lumen_ remain unknown. The anomalous subdiffusion of cytoplasmic Ca^2+^ and the random distribution of JSR RyRs, unnoticed in these models, could unveil the temporal and spatial properties of Ca^2+^ sparks and quarks.

The objective of the study is to develop a mathematical model of JSR Ca^2+^ release to quantify the interplay of rogue and clustered RyRs for a detailed explanation of spontaneous Ca^2+^ sparks and quarks in cardiac myocytes under physiological conditions. A mathematical model was proposed to simulate the temporal and two-dimensional (2D) spatial distributions of Ca^2+^ sparks and quarks in a cardiac myocyte with consideration of the distribution of clustered and rogue RyRs on the JSR membrane and the anomalous subdiffusion of Ca^2+^ in the cytoplasm [19]. The model could explain various Ca^2+^ release events from a JSR and predict the firing probability of clustered RyRs activated by Ca^2+^ release through rogue RyRs. The stochastic opening of rogue and clustered RyRs was regulated by free Ca^2+^ concentrations in both cytoplasm and JSR lumen of a cardiac myocyte. The line-scan experimental measurements were carried out in cardiac myocytes of rats to validate the present model. The significance was discussed to improve the understanding of Ca^2+^-induced-Ca^2+^-release events in cardiac myocytes.

## 2. Material and methods

### 2.1. Experimental methods

Similar to previous studies [20–22], eight Sprague-Dawley rats (about 2.5 months old, 225–300 g) were anaesthetized with pentobarbital sodium (40 mg kg^−1^) by peritoneal injection. Hearts were rapidly excised from animals. An isolated heart was immediately put into ice cold buffer, mounted in a Langendorff system, and perfused with a Ca^2+^-free buffer containing (in mM): 137 NaCl, 5.4 KCl, 1.2 MgCl_2_, 1.2 Na_2_HPO_4_, 20 HEPES, 10 taurine, and 10 glucose (at a pH value of 7.35, aerated with 95% O_2_ and 5% CO_2_) for 5 min. The heart was then digested in a buffer containing 0.5 mg ml^−1^ collagenase, 1 mg ml^−1^ bovine serum albumin, 0.06 mg ml^−1^ protease (type X-IV) and 50 µM CaCl_2_ until becoming pale. The left ventricle was sectioned into small pieces and incubated in the digesting solution. Myocytes were harvested and stored in Tyrode's solution containing (in mM): 135 NaCl, 1 CaCl_2_, 4 KCl, 1.2 MgCl_2_, 1.2 Na_2_HPO_4_, 10 glucose, and 10 HEPES with a pH value of 7.35. Before imaging, myocytes were loaded with the dye Fluo-4-AM for 5 min and washed twice using the Tyrode's solution. A confocal microscope (Nikon A1+, Japan) equipped with a 40 × 1.3 NA oil immersion objective was used for line-scan images at a sample rate of 512 frames s^−1^. The samples were excited at 488 nm. All line-scan measurements were performed at room temperature (23–25°C). Images were processed by the SparkMaster software [23].

### 2.2. Geometrical model

**Figure 1.**
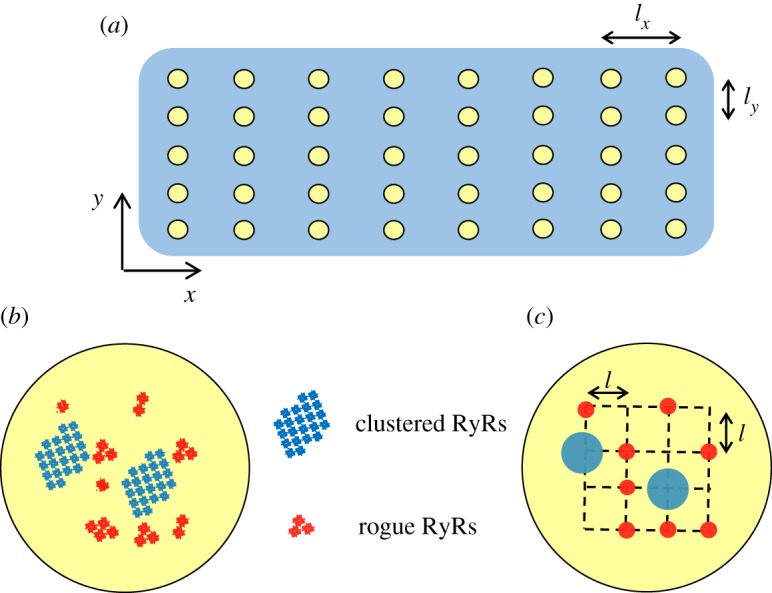
Geometrical model. (*a*) Schematic representation of the 2D geometrical model of a cardiac myocyte. The yellow dots denote JSRs with regularly spaced intervals: *l_x _*= 2 µm and *l_y_* = 0.8 µm. (*b*) Schematic representation of a JSR, which includes randomly distributed clustered and rogue RyRs. (*c*) The distribution of clustered and rogue RyRs in a JSR for simulation (*l* = 0.1 µm). Two CRUs of clustered RyRs (blue dots) are surrounded by eight rogue RyRs (red dots) on the JSR as shown in (*b*). The number and location of clustered and rogue RyRs are random in simulations.

In a 2D model similar to Izu *et al*. [24], the regular intervals between Ca^2+^ release sites are *l_x_* (= 2 µm) in the longitudinal direction (*x*-axis) and *l_y_* (= 0.8 µm) in the transverse direction (*y*-axis). Figure 1*a* shows the geometrical model of a cardiac myocyte. Each Ca^2+^ release site represents a JSR. The schematic representative of a JSR is shown in figure 1*b*, which includes randomly distributed clustered RyRs and rogue RyRs. A CRU of clustered RyRs has 22 RyR channels and a rogue RyR has 3 RyR channels [5]. Figure 1*c* shows the distribution of clustered and rogue RyRs on a JSR for simulation. CRUs of clustered RyRs (blue dots, approx. 2 in a JSR) are surrounded by randomly distributed rogue RyRs (red dots, approx. 8 in a JSR). The number and location of clustered and rogue RyRs in each JSR are random in simulations.

### 2.3. Computational model

The balanced equation for free Ca^2+^ concentration in the cytoplasm, ${\left[\text{C}{\text{a}}^{2+}\right]}_{\mathrm{cyto}}$, with consideration of the anomalous subdiffusion of Ca^2+^ in the cytoplasm and the distribution of clustered and rogue RyRs, can be written as:

2.1 $$\begin{array}{rl} \frac{\partial {\left[\text{C}{\text{a}}^{2+}\right]}_{\mathrm{cyto}}}{\partial t} & =D_{x}\frac{\partial^{\beta }{\left[\text{C}{\text{a}}^{2+}\right]}_{\mathrm{cyto}}}{\partial x^{\beta }}+D_{y}\frac{\partial^{\beta }{\left[\text{C}{\text{a}}^{2+}\right]}_{\mathrm{cyto}}}{\partial y^{\beta }}\\ & \;+J_{\mathrm{dye}\text{-}\mathrm{cyto}}+J_{\mathrm{buffer}\text{-}\mathrm{cyto}}+J_{\mathrm{pump}}+J_{\mathrm{clustered}}+J_{\mathrm{rogue}}, \end{array}$$

where *t* is time, *x* and *y* denote the spatial coordinates, *D*_x_ (= 300 µm^2^ s^-1^) and *D*_y_ (= 150 µm^2^ s^−1^) refer to the Ca^2+^ diffusion coefficients. The anomalous subdiffusion order *β* is 2.25. Anomalous space subdiffusion corresponds to the short jump of the random walker and is defined though the relations [25]

2.2 $$\frac{\partial^{2.25}{\left[\text{C}{\text{a}}^{2+}\right]}_{\mathrm{cyto}}(x,y,t)}{\partial x^{2.25}}=\frac{1}{\Gamma (0.75)}\frac{\partial^{3}}{\partial x^{3}}\int_{0}^{x}\frac{{\left[\text{C}{\text{a}}^{2+}\right]}_{\mathrm{cyto}}(\tau ,y,t)}{{\left(x-\tau \right)}^{0.25}}\text{d}\tau$$

and

2.3 $$\frac{\partial^{2.25}{\left[\text{C}{\text{a}}^{2+}\right]}_{\mathrm{cyto}}(x,y,t)}{\partial y^{2.25}}=\frac{1}{\Gamma (0.75)}\frac{\partial^{3}}{\partial y^{3}}\int_{0}^{y}\frac{{\left[\text{C}{\text{a}}^{2+}\right]}_{\mathrm{cyto}}(x,\tau ,t)}{{\left(y-\tau \right)}^{0.25}}\text{d}\tau ,$$

where *Γ* denotes the Gamma function. $J_{\mathrm{dye}\text{-}\mathrm{cyto}}$ and $J_{\mathrm{buffer}\text{-}\mathrm{cyto}}$ are the fluxes due to the Ca^2+^ fluorescent indicator dye (i.e. Rhod-2 or Fluo-4-AM, in the cytoplasm) and the endogenous stationary buffers respectively. *J*_pump_ is the pumping rate of SR Ca^2+^-ATPase. SR pumps are started when ${\left[\text{C}{\text{a}}^{2+}\right]}_{\mathrm{cyto}}$ exceeds the resting Ca^2+^ concentration level (0.1 µM) in the cytoplasm. These variables can all be defined as:

2.4 $$\begin{array}{r} J_{\mathrm{dye}\text{-}\mathrm{cyto}}=-k_{F}^{+}{\left[\text{C}{\text{a}}^{2+}\right]}_{\mathrm{cyto}}({\left[\text{F}\right]}_{T}-[\text{CaF}])+k_{F}^{-}[\text{CaF}], \end{array}$$

2.5 $$\begin{array}{r} J_{\mathrm{buffer}\text{-}\mathrm{cyto}}=\sum_{n}-\frac{\partial [\text{Ca}{\text{B}}_{n}]}{\partial t}, \end{array}$$

2.6 $$\begin{array}{r} \frac{\partial [\text{Ca}{\text{B}}_{n}]}{\partial t}=k_{n}^{+}{\left[\text{C}{\text{a}}^{2+}\right]}_{\mathrm{cyto}}({\left[\text{B}\right]}_{T}-[\text{Ca}{\text{B}}_{n}])-k_{n}^{-}[\text{Ca}{\text{B}}_{n}] \end{array}$$

2.7 $$\begin{array}{r} \;\text{and}\;J_{\mathrm{pump}}=-\frac{V_{\mathrm{pump}}^{\max}{\left({\left[\text{C}{\text{a}}^{2+}\right]}_{\mathrm{cyto}}\right)}^{h}}{{\left(K_{\mathrm{pump}}\right)}^{h}+{\left({\left[\text{C}{\text{a}}^{2+}\right]}_{\mathrm{cyto}}\right)}^{h}}, \end{array}$$

where the subscript ‘n’ refers to each buffer in the cytoplasm, the superscript ‘h’ refers to the Hill constant; [F]_T_ and [B_n_]_T_ represent the total concentrations of indicator and buffers, respectively. [CaF] and [CaB_n_] are the concentrations of Ca^2+^-bound complexes; $k_{F}^{+}$, $k_{F}^{-}$, $k_{n}^{+}$ and $k_{n}^{-}$ are the reaction kinetic parameters. *K*_pump_ is the affinity constant, and $V_{\mathrm{pump}}^{\max}$ is the maximum rate for SR pumps. Values of the parameters are based on a previous study [26]. Moreover, *J*_rogue_ and *J*_clustered_ are the Ca^2+^ fluxes released by rogue and clustered RyRs respectively, which can be written as:

2.8 $$J_{\mathrm{rogue}}=\sigma_{\mathrm{rogue}}\sum_{i,j}\delta (x-x_{i}^{\mathrm{rogue}},y-y_{j}^{\mathrm{rogue}})S(x_{i}^{\mathrm{rogue}},y_{j}^{\mathrm{rogue}},t;\;T_{\mathrm{rogue}})$$

and

2.9 $$J_{\mathrm{clustered}}=\sigma_{\mathrm{clustered}}\sum_{i,j}\delta (x-x_{i}^{\mathrm{clustered}},y-y_{j}^{\mathrm{clustered}})S(x_{i}^{\mathrm{clustered}},y_{j}^{\mathrm{clustered}},t;\;T_{\mathrm{clustered}}),$$

where *δ* is the Dirac delta function and *S* is a stochastic function for the opening of clustered and rogue RyRs; $\left(x_{i}^{\mathrm{rogue}},\;y_{j}^{\mathrm{rogue}}\right)$ and $\left(x_{i}^{\mathrm{clustered}},\;y_{j}^{\mathrm{clustered}}\right)$are the positions of rogue RyRs and clustered RyRs in the 2D plane respectively. The release times for rogue and clustered RyRs are defined as *T*_rogue_= 20 ms [10] and *T*_clustered_= 10 ms [27]. The equivalent source strength of rogue RyRs and clustered RyRs [28] are expressed by

2.10 $$\sigma_{\mathrm{rogue}}=\frac{0.64I_{\mathrm{rogue}}({\left[\text{C}{\text{a}}^{2+}\right]}_{\mathrm{lumen}}-{\left[\text{C}{\text{a}}^{2+}\right]}_{\mathrm{cyto}})}{2F}$$

and

2.11 $$\sigma_{\mathrm{clustered}}=\frac{0.64I_{\mathrm{clustered}}({\left[\text{C}{\text{a}}^{2+}\right]}_{\mathrm{lumen}}-{\left[\text{C}{\text{a}}^{2+}\right]}_{\mathrm{cyto}})}{2F},$$

where the Faraday constant *F* is 96 500 C mol^−1^, and *I*_rogue_ and *I*_clustered_ are the average currents through rogue and clustered RyRs, set to be 0.07 pA mM^−1^ and 0.7 pA mM^−1^. Note that Ca^2+^ is released from the JSR lumen into a 3D volume in the cytoplasm of a cardiac myocyte. The conversion factor 0.64 in equations (2.10) and (2.11) is used to give the identical Ca^2+^ distribution in 2D [24]. Equation (2.1) can describe the Ca^2+^ release mechanism of Ca^2+^ sparks only when *J*_rogue_ = 0, or QCRs only when *J*_clustered_ = 0.

Conversely, the balance equation for free Ca^2+^ concentration in each JSR lumen, ${\left[\text{C}{\text{a}}^{2+}\right]}_{\mathrm{lumen}}$, can be written as

2.12 $$\frac{\partial {\left[\text{C}{\text{a}}^{2+}\right]}_{\mathrm{lumen}}}{\partial t}=J_{\mathrm{release}\text{-}\mathrm{lumen}}+J_{\mathrm{dye}\text{-}\mathrm{lumen}}+J_{\mathrm{buffer}\text{-}\mathrm{lumen}}+J_{\mathrm{refill}},$$

where $J_{\mathrm{release}\text{-}\mathrm{lumen}}$ denotes the decreased Ca^2+^ release flux caused by opening of clustered RyRs (*J*_clustered_) and rogue RyRs (*J*_rogue_) in a JSR. *J*_refill_ is the refilled Ca^2+^ flux and expressed by

2.13 $$J_{\mathrm{refill}}=\frac{{\left[\text{C}{\text{a}}^{2+}\right]}_{\mathrm{NSR}}-{\left[\text{C}{\text{a}}^{2+}\right]}_{\mathrm{lumen}}}{\tau_{\mathrm{refill}}},$$

where free Ca^2+^ concentration in network sarcoplasmic reticulum (NSR) [Ca^2+^]_NSR_ is 1.0 mM, time constant for Ca^2+^ transfer between JSR and NSR $\tau_{\mathrm{refill}}$ is 10 ms, and the volume of a JSR lumen is 1 × 10^−11^ µl [29]. [Ca^2+^]_lumen_ is less than the beginning level 1.0 mM as a result of Ca^2+^ release at a certain time. $J_{\text{dye-lumen}}$ and $J_{\text{buffer-lumen}}$ are the Ca^2+^ fluxes due to indicator dye (i.e. Fluo-5N) and buffer (i.e. calsequestrin) in a JSR lumen, respectively. Their expressions are similar to that in the cytoplasm. Various parameters of dyes (Rhod-2 [30], Fluo-4-AM [26] and Fluo-5N [31]) and buffers [26,32] in the cytoplasm and JSR lumen are listed in table 1.

**Table 1.** Standard parameter values for dyes and buffers.

dyes or buffers	[F]_T_ or [B_n_]_T_ (μM)	*k*_F_^+^ or *k*_n_^+^ (μM^−1^ s^−1^)	*k*_F_^−^ or *k*_n_^−^ (s^−1^)
parameters in cytoplasm			
Rhod-2	5	130	69
Fluo-4-AM	50	80	90
calmodulin	24	100	38
troponin	70	39	20
SR	47	115	100
SL	1124	115	1000
parameters in JSR lumen			
Fluo-5N	20	48.8	19 520
calsequestrin	14 000	100	60 000

### 2.4. Firing probability of rogue and clustered RyRs

The firing probability per unit time of RyRs is determined by Ca^2+^ concentrations in both cytoplasm and JSR lumen [33–35], which can be expressed as

2.14 $$P_{\mathrm{firing}}=P_{\mathrm{cyto}}\cdot \Phi_{\mathrm{lumen}},$$

where *P*_cyto_ refers to the firing probability per unit time of calcium release events controlled by ${\left[\text{C}{\text{a}}^{2+}\right]}_{\mathrm{cyto}}$. $\Phi_{\mathrm{lumen}}$ represents a [Ca^2+^]_lumen_-dependent regulation term of Ca^2+^ release events. According to the coupled RyR gating model [5], *P*_cyto_ can be expressed as

2.15 $$P_{\mathrm{cyto}}=1-{\left(1-P{}_{\mathrm{RyR}}\right)}^{n{}_{\mathrm{RyR}}},$$

where $P_{\mathrm{RyR}}$ is the firing probability per unit time of a single RyR channel [36]. Here, $\Phi_{\mathrm{lumen}}$ is written as

2.16 $$\Phi_{\mathrm{lumen}}=\phi^{m},$$

where *ϕ* is an empirical power function given in Walker *et al*.'s model [14], *m* is the regulation coefficient for rogue (*m *= 1) or clustered (*m* = 10) RyRs.

### 2.5. Numerical solutions

Equations (2.1–2.16) were solved using a FORTRAN-developed program. A 2D computational domain (5 µm × 5 µm) was meshed with a size of 0.025 µm to simulate Ca^2+^ release events from a single Ca^2+^ release site. Moreover, a computational domain of 20 µm × 20 µm was meshed with a size of 0.1 µm to simulate Ca^2+^ release events from multiple Ca^2+^ release positions. For the fractional differential term in equation (2.1), the shifted Grünwald formula of centre difference [37] was used to discretize the computational domain as

2.17 $$\frac{\partial^{\alpha }{\left[\text{C}{\text{a}}^{2+}\right]}_{\mathrm{cyto}}(x,y,t)}{\partial x^{\alpha }}=\frac{1}{h^{\alpha }}\underset{M\to \infty }{\lim}\sum_{k=0}^{M}g_{k}{\left[\text{C}{\text{a}}^{2+}\right]}_{\mathrm{cyto}}(x-(k-1)h,y,t)$$

and

2.18 $$\frac{\partial^{\alpha }{\left[\text{C}{\text{a}}^{2+}\right]}_{\mathrm{cyto}}(x,y,t)}{\partial y^{\alpha }}=\frac{1}{h^{\alpha }}\underset{M\to \infty }{\lim}\sum_{k=0}^{M}g_{k}{\left[\text{C}{\text{a}}^{2+}\right]}_{\mathrm{cyto}}(x,y-(k-1)h,t),$$

where $g_{k}=\Gamma (k-\alpha )/\Gamma (k+1)$, *α* = *β *− 1 = 1.25, *k* is an integer with *α < k < α *+ 1, and *h* is the mesh size. Free Ca^2+^ concentrations in the cytoplasm and JSR were calculated simultaneously. The variable time-step algorithm was used. The zero-flux boundary condition was taken in the Monte Carlo simulations.

## 3. Results and discussion

### 3.1. Ca^2+^ quarks and Ca^2+^ sparks

**Figure 2.**
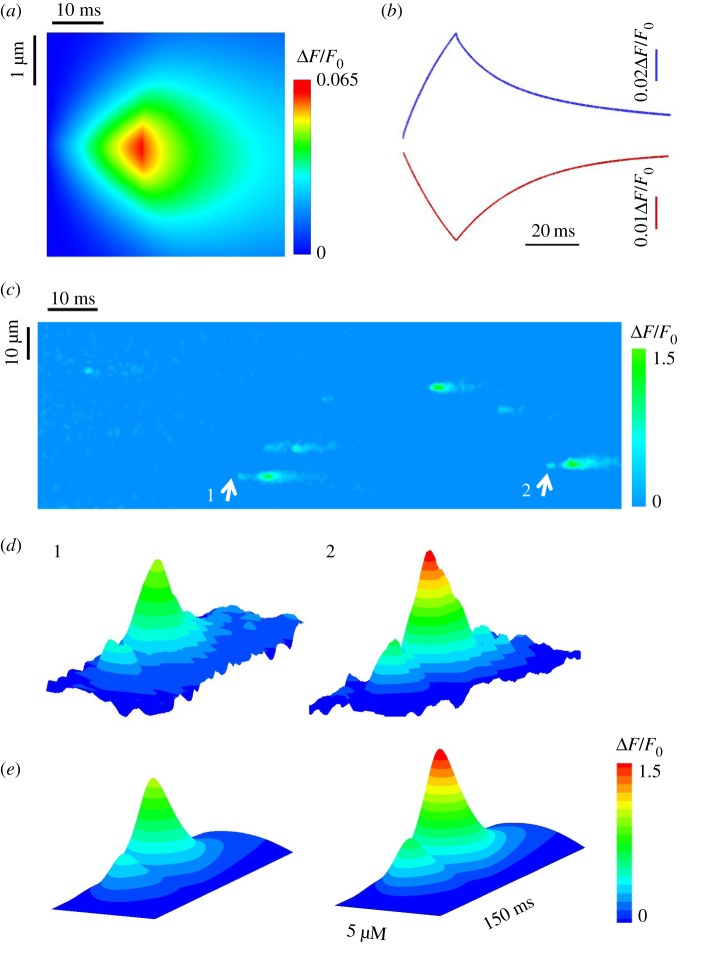
Properties of Ca^2+^ quarks and Ca^2+^ sparks. (*a*) A computational line-scan Ca^2+^ quark through a rogue RyR (5 µm × 50 ms). (*b*) The corresponding time courses of a QCR–QCD pair. (*c*) A representative line-scan image (50 µm × 1 s) of Ca^2+^ release events measured in a cardiac myocyte. Arrows point to Ca^2+^ sparks activated by QCRs. (*d*) Experimental results for line-scan images of sparks activated by QCRs. (*e*) Computational results in agreement with (*d*).

Figure 2*a* shows a computational Ca^2+^ quark through a rogue RyR to mimic the line-scan measurements when the release time is set to 20 ms. The computational domain is a square of 5 × 5 µm^2^ with the distribution of clustered and rogue RyRs on the JSR membrane in figure 1*c*. The dyes, Rhod-2 and Fluo-5N, were used to indicate Ca^2+^ in the cytoplasm and JSR lumen, respectively. The shape of the Ca^2+^ quark is consistent with that in a previous study [10]. Figure 2*b* plots the time courses of a QCR–QCD pair (i.e. a quarky Ca^2+^ release–quarky Ca^2+^ depletion pair) corresponding to figure 2*a*. The values of *t*_67_ and Δ*F*/*F*_0_ were computed to be 22.0 and 22.5 ms and 0.065 and 0.025 for QCR and QCD, respectively. They are within 1 s.d. of experimental measurements, i.e. *t*_67_ = 20.1 ± 1.1 ms for a QCR and 20.8 ± 1.9 ms for a QCD and Δ*F*/*F*_0_ = 0.069 ± 0.006 for a QCR and 0.025 ± 0.002 for a QCD [10].

Figure 2*c* shows the line-scan measurements of Ca^2+^ release events in an isolated myocyte. The arrows refer to Ca^2+^ sparks due to the firing of clustered RyRs after QCR events owing to the opening of rogue RyRs, which were further analysed using the SparkMaster software [23] in figure 2*d*. The peak Δ*F*/*F*_0_ of the two Ca^2+^ spark is 1.02 and 1.44 in figure 2*d* (1) and (2), respectively. To avoid the background noise, we did not measure QCR events with Δ*F*/*F*_0_ < 0.2. The number of recorded Ca^2+^ sparks is 1125 in all measurements. The proportion of sparks that are triggered by QCRs is approximately 11.6%. Accordingly, figure 2*e* shows the computational results of Ca^2+^ sparks in a JSR with random distribution of clustered and rogue RyRs in figure 1*c*. The regular Ca^2+^ spark (left) due to an opening CRU of clustered RyRs is initiated by three opening rogue RyRs. The large spark (right) results from two CRUs of clustered RyRs activated by four opening rogue RyRs. An agreement between experimental and computational results (figure 2*d* versus figure 2*e*) validates the 2D mathematical model.

### 3.2. Interplay of rogue and clustered RyRs in a junctional sarcoplasmic reticulum

**Figure 3.**
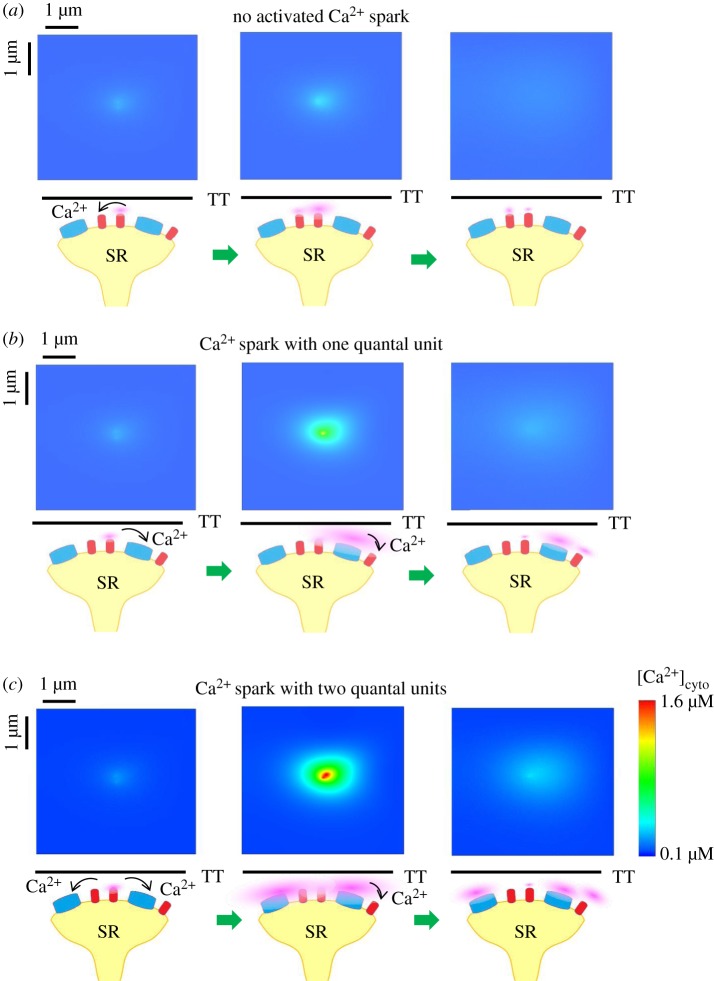
Interplay of rogue and clustered RyRs in a JSR. Snapshots of Ca^2+^ release events in a region of 5 µm × 5 µm are taken at 10, 20 and 40 ms from left to right when three rogue RyRs are fired at the same time. There are three distinct modes. Schematic interplay of Ca^2+^ release events is plotted below the snapshots. Clustered and rogue RyRs are distinguished by blue and red colours similar to figure 1*c*. Cytoplasmic Ca^2+^ is displayed by pink colour. Arrows denote that Ca^2+^ diffuses and activates neighbour clustered and rogue RyRs in the cytoplasm. SR, sarcoplasmic reticulum; TT, T-tubule. (*a*) No activated Ca^2+^ spark. Neighbour rogue RyRs are activated by Ca^2+^ quarks. (*b*) A Ca^2+^ spark with one quantal unit. One CRU of clustered RyRs is initiated by Ca^2+^ quarks. Then Ca^2+^ spark from the CRU of clustered RyRs contributes to activation of neighbour rogue RyRs. (*c*) A Ca^2+^ spark with two quantal units. Two CRUs of clustered RyRs are triggered and then activate neighbour rogue RyRs.

We simulated three modes of elemental Ca^2+^ release events that could coexist at a Ca^2+^ release site, as shown in figure 3*a–c*. Snapshots of elemental Ca^2+^ release events in a computational domain of 5 × 5 µm^2^ are taken at 10, 20 and 40 ms when three rogue RyRs are fired at the same time. Given the polymorphism of Ca^2+^ sparks at a release site, there are three distinct modes: no activated Ca^2+^ spark, a Ca^2+^ spark with one quantal unit (i.e. a fired Ca^2+^ spark from a CRU of clustered RyRs), and a Ca^2+^ spark with two quantal units (i.e. a fired Ca^2+^ spark from two CRUs of clustered RyRs). It demonstrates that QCRs from the opening rogue RyRs could activate the neighbour CRUs of clustered RyRs in a JSR to form a Ca^2+^ spark with different quantal units. Moreover, rogue RyRs could be activated by the Ca^2+^ release of clustered RyRs in the same JSR, which was recorded in the snapshots and shown in figure 3*b*,*c*. On the other hand, the amplitude of a Ca^2+^ spark is mainly determined by the firing number of clustered RyRs regardless of rogue RyRs because QCR events have a low Ca^2+^ flux.

**Figure 4.**
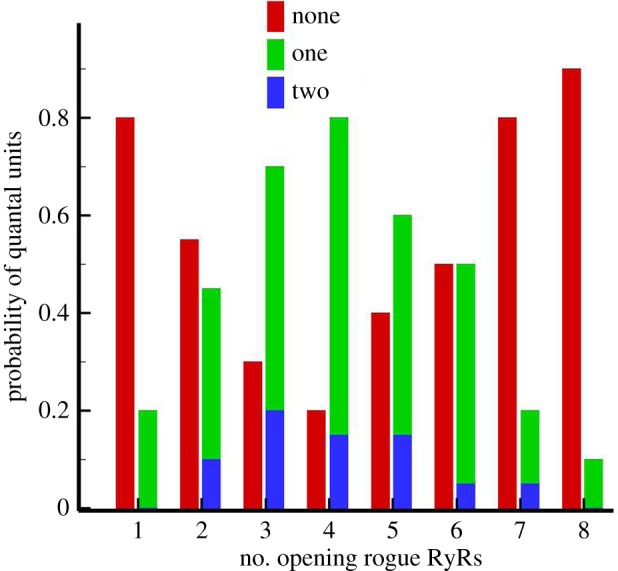
The probability of activated quantal units in a JSR calculated by Monte Carlo simulations (*n* = 20).

This model explains the coexistence of the three modes of elemental Ca^2+^ release in a Ca^2+^ release site as well as predicting the properties of a Ca^2+^ spark. QCRs do not always activate clustered RyRs because luminal Ca^2+^ depletion reduces the probability of activating clustered RyRs. The probability of Ca^2+^ sparks of the three modes activated by different initial opening numbers of rogue RyRs is shown in figure 4. The results reveal that clustered RyRs have the highest frequency of being triggered by four opening rogue RyRs simultaneously.

### 3.3. The effects of [Ca^2+^]_lumen_ on Ca^2+^ release events

**Figure 5.**
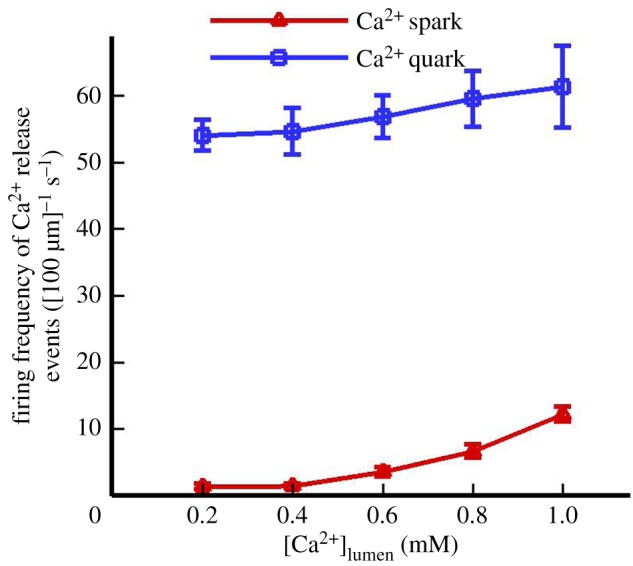
The firing frequency of Ca^2+^ sparks (red line) and quarks (blue line) determined by Monte Carlo simulations (*n* = 10) when [Ca^2+^]_lumen_ varies from 0.2 mM to 1.0 mM.

The effects of [Ca^2+^]_lumen_ on the firing frequency of Ca^2+^ sparks and quarks are quantified in a computational domain of 20 × 20 µm^2^, as shown in figure 5. The beginning levels of [Ca^2+^]_lumen_ is set from 0.2 to 1.0 mM. The computational results show a higher firing frequency of spontaneous QCR events than that of spontaneous Ca^2+^ sparks consistent with previous experimental observations [10]. A computational study also showed a steep increase in the firing frequency of spontaneous Ca^2+^ sparks despite a slight change in the firing frequency of spontaneous QCR events with the increase of [Ca^2+^]_lumen_ [18]. Moreover, we show a threshold value of [Ca^2+^]_lumen_ (i.e. less than 0.3 mM) where spontaneous Ca^2+^ quarks become a major pathway of SR Ca^2+^ leak. The present study shows the incidence of 12.2 ± 1.1 Ca^2+^ sparks [100 µm]^−1^ s^−1^ under physiological conditions ([Ca^2+^]_lumen_ = 1.0 mM), which agrees with experimental measurements [38]. The statistical proportion of sparks that are triggered by QCRs is 34.7% because one or two openings of rogue RyRs were neglected in experimental measurements in figure 2*c*.

### 3.4. Effects of rogue RyRs on Ca^2+^ sparks with consideration of [Ca^2+^]_lumen_

**Figure 6.**
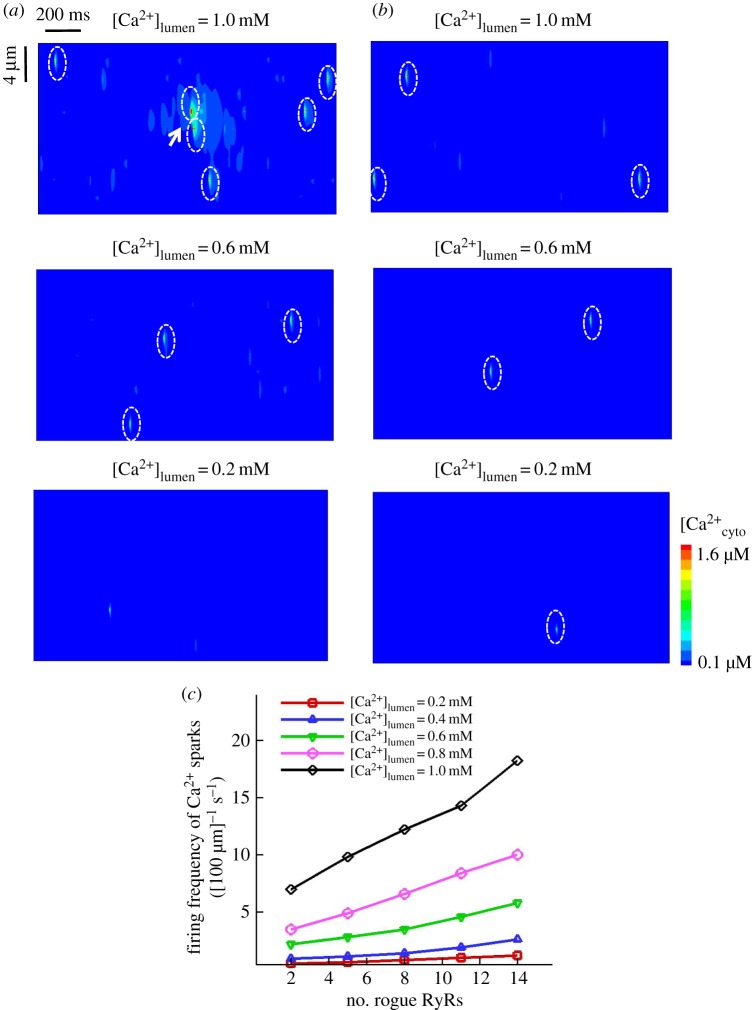
Effects of rogue RyRs on Ca^2+^ sparks as [Ca^2+^]_lumen_ varies. (*a*) Computational line-scan Ca^2+^ release events (20 µm × 2 s) with effects of rogue RyRs when [Ca^2+^]_lumen_ = 1.0 mM, 0.6 mM and 0.2 mM. Ca^2+^ sparks are marked by white ovals. (*b*) Computational line-scan Ca^2+^ release events without effects of rogue RyRs under the same conditions as (*a*). (*c*) Sensitivity analysis on the firing frequency of Ca^2+^ sparks as a function of the number of rogue RyRs in a JSR at various [Ca^2+^]_lumen_ determined by Monte Carlo simulations (*n* = 10).

We examined how rogue RyRs affect Ca^2+^ sparks at different levels of [Ca^2+^]_lumen_. A comparison of computational line-scan Ca^2+^ release events with consideration of rogue RyRs or not is displayed in a square of 20 × 20 µm^2^ (the intervals between JSRs are *l_x _*= 2 µm and *l_y _*= 0.8 µm) for 2 s (figure 6*a* versus figure 6*b*) at different [Ca^2+^]_lumen_. The Ca^2+^ spark frequency has values of 1.3 ± 0.4 and 1.2 ± 0.3 [100 µm]^−1^ s^−1^ with consideration of rogue RyRs or not when [Ca^2+^]_lumen_ = 0.2 mM. Therefore, the stochastic opening of rogue RyRs at low [Ca^2+^]_lumen_ fails to trigger spontaneous Ca^2+^ sparks owing to the decreased driving force ([Ca^2+^]_lumen _− [Ca^2+^]_cyto_) and sensitivity of clustered RyRs as well as the shortened firing possibility of neighbouring RyRs. Conversely, the frequency of spontaneous Ca^2+^ sparks increases with consideration of rogue RyRs at 1.0 mM [Ca^2+^]_lumen_. QCR events are hence responsible for the formation of Ca^2+^ sparks at high [Ca^2+^]_lumen_. Moreover, sensitivity analysis on the firing frequency of Ca^2+^ sparks was performed with respect to the number of rogue RyRs varying in the range of 2–14 in figure 6*c*. The opening of clustered RyRs monotonically increases with the increase of the number of rogue RyRs at high [Ca^2+^]_lumen_. But it does not change obviously at low [Ca^2+^]_lumen_. The firing frequency of Ca^2+^ sparks has a bigger slope at higher [Ca^2+^]_lumen_.

### 3.5. A comparison with previous models

Sato *et al*. [18] showed that a RyR channel in a cluster of RyRs could trigger adjacent RyR channels in the same cluster to form a Ca^2+^ spark when [Ca^2+^]_lumen_ is above a threshold. Walker *et al*. [14,15] further investigated the structural effects of clustered RyRs on Ca^2+^ sparks. Moreover, Lu *et al*. [16,17] indicated that non-junctional RyRs increased the probability of occurrence of spontaneous Ca^2+^ waves. The present study showed the temporal and spatial properties of Ca^2+^ quarks and sparks relevant to both rogue and clustered RyRs, where rogue RyRs randomly surround clustered RyRs within a single junctional space. We showed that Ca^2+^ quarks and sparks coexist at a Ca^2+^ release site, which agrees with experimental measurements from line-scan imaging. The opening of rogue RyRs leads to the formation of Ca^2+^ sparks at high [Ca^2+^]_lumen_, but not at low [Ca^2+^]_lumen_. This supports the conclusion of Sato *et al*. [18].

On the other hand, the present model simulated Ca^2+^ quarks and sparks based on the anomalous subdiffusion in comparison with previous models from Fickian diffusion [14–18]. Hence, we solved the paradox of full width at half-maximum (FWHM) due to Fickian diffusion. This study addresses the importance of rogue RyRs for understanding Ca^2+^ release mechanisms from JSRs.

### 3.6. Potential implications

**Figure 7.**
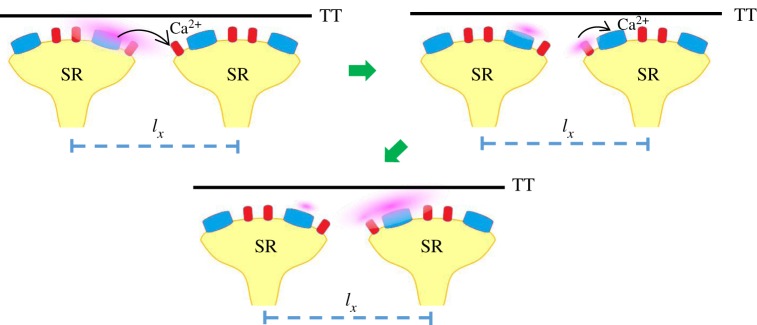
Schematic interplay of clustered and rogue RyRs in neighbour JSRs. Clustered RyRs are triggered by Ca^2+^ sparks in a neighbour JSR with the help of rogue RyRs.

Ca^2+^ sparks could trigger clustered RyRs in neighbour JSRs with the help of rogue RyRs. This mode is marked by the arrow in figure 6*a* and shown in figure 7 schematically. Ca^2+^ quarks may trigger the opening of clustered RyRs in self-propagating succession along the length of a cell. The sum of Ca^2+^ sparks and quarks gives rise to the global Ca^2+^ transient for the formation of a Ca^2+^ wave. Furthermore, the changes in the number of rogue RyRs in a JSR may induce potential heart diseases. For example, a reduction of the number of rogue RyRs could lead to an inhibition of Ca^2+^ waves and dyssynchronous Ca^2+^ transients in myocytes of congestive heart failure [39]. Atrial fibrillation associated with overactive Ca^2+^ release could be related to the increased number of rogue RyRs [40].

### 3.7. Critique of the study

In the study, the duration and current of Ca^2+^ release events from JSRs were fixed similar to previous studies [10,27]. However, Ca^2+^ release flux should be regulated by the SR structure, functional properties and the size of RyR cluster [41]. The impact of time-dependent Ca^2+^ release flux from RyRs can give us new inspiration for the relation between Ca^2+^ release events and the interplay of rogue and clustered RyRs. The spatial arrangement of RyRs within clusters influences the frequency of Ca^2+^ sparks [14]. The detailed structure of clustered RyRs should be taken into consideration when a high-performance supercomputer is used to satisfy the requirement of large computation. Furthermore, the present study comes from the assumption that 3D geometry is simplified to a 2D model in healthy myocytes. Modelling 3D distribution of the JSRs in cardiac cells is more realistic and the 3D simulations of Izu *et al.* [42] indicated that it could reveal more complex RyR interactions between neighbour JSRs. Hence, a 3D model should be developed to investigate spontaneous Ca^2+^ release events under both physiological and pathological conditions in future studies.

## 4. Conclusion

A mathematical model is developed to investigate Ca^2+^ sparks and quarks in the cytoplasm and show the significance of rogue RyRs. The Ca^2+^ release events from JSRs agree with experimental measurements in cardiac myocytes. The computational results show a steep increase in the firing frequency of spontaneous Ca^2+^ sparks despite a slight change in the firing frequency of spontaneous Ca^2+^ sparks with the increase of [Ca^2+^]_lumen_. The frequency of spontaneous Ca^2+^ sparks is remarkably affected by the rogue RyRs opening at high [Ca^2+^]_lumen_, but not at low [Ca^2+^]_lumen_. This study is of importance to understand basic mechanisms of Ca^2+^ release events in cardiac myocytes.

## Data accessibility

Statistical data of Ca^2+^ release events used in this paper can be accessed at: http://dx.doi.org/10.6084/m9.figshare.5450719.
